# A distributed ASTRA toolbox

**DOI:** 10.1186/s40679-016-0032-z

**Published:** 2016-12-07

**Authors:** Willem Jan Palenstijn, Jeroen Bédorf, Jan Sijbers, K. Joost Batenburg

**Affiliations:** 1 CWI, Amsterdam, The Netherlands; 2 Leiden Observatory, Universiteit Leiden, Leiden, The Netherlands; 3 iMinds-Vision Lab, Antwerp University, Antwerp, Belgium; 4 Mathematisch Instituut, Universiteit Leiden, Leiden, The Netherlands

**Keywords:** Tomography, Reconstruction, Distributed computation

## Abstract

While iterative reconstruction algorithms for tomography have several advantages compared to standard backprojection methods, the adoption of such algorithms in large-scale imaging facilities is still limited, one of the key obstacles being their high computational load. Although GPU-enabled computing clusters are, in principle, powerful enough to carry out iterative reconstructions on large datasets in reasonable time, creating efficient distributed algorithms has so far remained a complex task, requiring low-level programming to deal with memory management and network communication. The ASTRA toolbox is a software toolbox that enables rapid development of GPU accelerated tomography algorithms. It contains GPU implementations of forward and backprojection operations for many scanning geometries, as well as a set of algorithms for iterative reconstruction. These algorithms are currently limited to using GPUs in a single workstation. In this paper, we present an extension of the ASTRA toolbox and its Python interface with implementations of forward projection, backprojection and the SIRT algorithm that can be distributed over multiple GPUs and multiple workstations, as well as the tools to write distributed versions of custom reconstruction algorithms, to make processing larger datasets with ASTRA feasible. As a result, algorithms that are implemented in a high-level conceptual script can run seamlessly on GPU-enabled computing clusters, up to 32 GPUs or more. Our approach is not limited to slice-based reconstruction, facilitating a direct portability of algorithms coded for parallel-beam synchrotron tomography to cone-beam laboratory tomography setups without making changes to the reconstruction algorithm.

## Background

In recent years, iterative reconstruction algorithms for tomography have demonstrated promising results in the ability to compute high-quality 3D images from less data compared to the classical backprojection algorithms [1–3]. Despite these results, the practical use of advanced iterative algorithms for X-ray tomography, in both synchrotron and laboratory settings, remains limited.

One of the key obstacles in the adoption of such algorithms is the requirements that it imposes on the hardware (computing and memory) and software (parallelization). Due to advances in modern X-ray cameras, experimental datasets and their corresponding 3D reconstructed volumes can easily occupy hundreds of gigabytes of computer memory. For classical backprojection methods, it is trivial to partition both computation and memory-usage into smaller portions that can each be processed independently. The computations can therefore be carried out on a distributed computing system (e.g. a large cluster) to reduce the computation time to acceptable levels [4, 5]. For iterative methods, however, such a decomposition is often not straightforward [6–8].

For single workstations, there are now many high-performance implementations of both classical backprojection methods and iterative methods, often using graphics processing units (GPUs), for both parallel and cone-beam geometries [9–14].

The main constraint when applying iterative reconstruction methods is that in many cases the full 3D volume must be loaded into computer memory at once during the reconstruction, such that the basic operations of forward projection (FP, computing the X-ray images for the given 3D volume) and backprojection (BP, the mathematical transpose of the forward projection) can both be carried out efficiently.

One notable exception to this memory requirement is tomography in a strictly parallel-beam illumination setting, using a single axis of rotation, which is common in synchrotron imaging. In this setting, each slice of the 3D volume is measured by a single row of the detector, allowing the reconstruction to be carried out independently for different slices. Although it is very powerful, this approach also has strong limitations. In particular, (1) small deviations from the ideal geometrical setup, such as slightly divergent X-ray beams or a slight tilt of the rotation axis, cannot be dealt with in slice-based algorithms; (2) to exchange algorithms between a synchrotron setup and the much more common cone-beam setups used in non-synchrotron X-ray labs, the entire algorithm must be recoded into a non-slice-based version; (3) many iterative algorithms make use of prior information about the object, which is often specified in 3D, thereby inducing dependencies between the reconstructions of different slices.

**Fig. 1:**
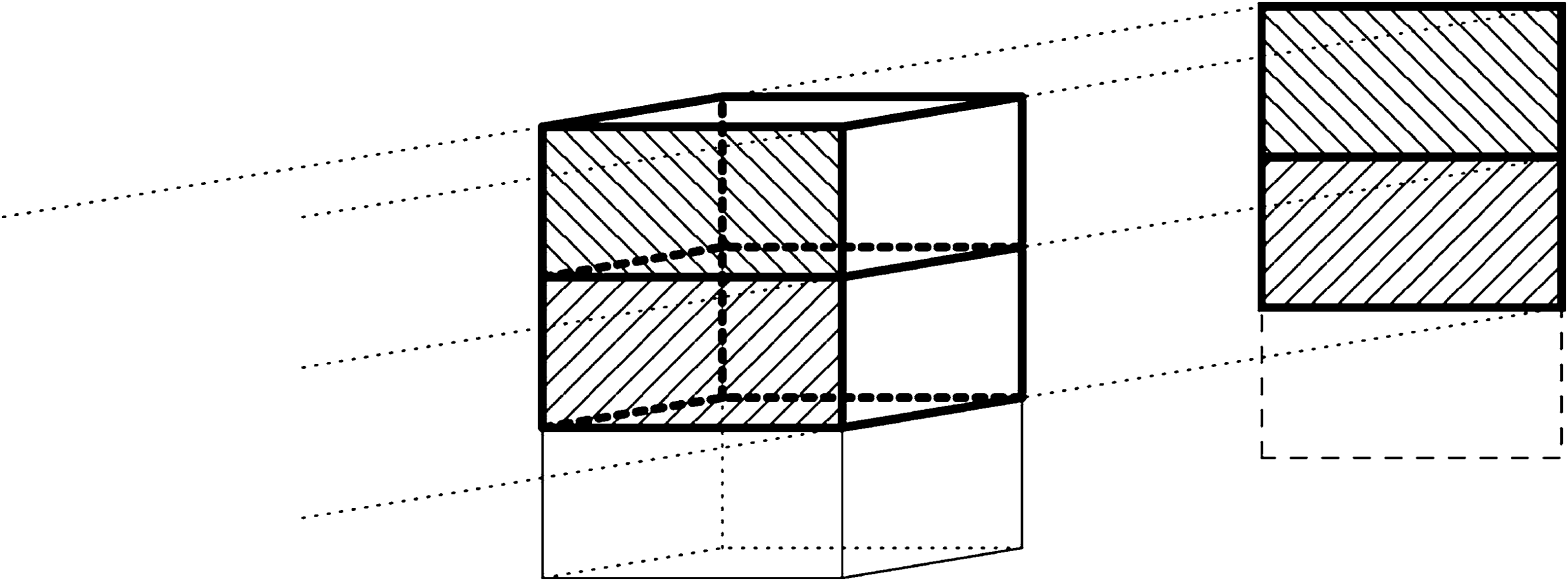
Parallel-beam projection of two volume slabs. It shows a parallel-beam projection of the cubic volume in the centre on the detector plane on the right. Two slabs in the volume are outlined in *black*, and indicated by North-West (NW) diagonal patterns and North-East (NE) diagonal patterns. The projections of these two slabs are correspondingly patterned with NW respectively NE diagonals, and do not overlap

**Fig. 2:**
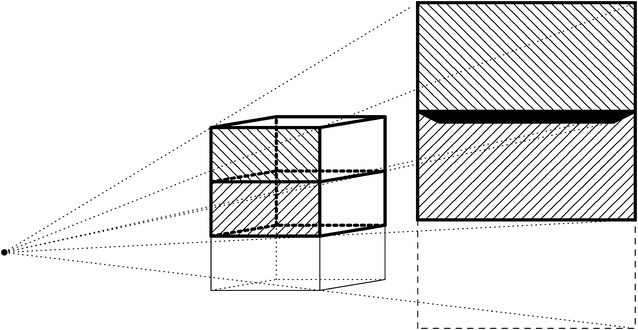
Cone-beam projection of two volume slabs. It shows a cone-beam projection of the cubic volume in the centre on the detector plane on the right. Two slabs in the volume are outlined in *black*, and indicated by North-West (NW) diagonal patterns and North-East (NE) diagonal patterns. The projections of these two slabs are correspondingly patterned with NW, respectively, NE diagonals. The *solidly filled area* shows where the projections of the two slabs overlap

The problem of performing large-scale iterative reconstructions on a distributed computing cluster is illustrated in Figs. 1 and 2. Figure 1 shows how the computations for a typical synchrotron tomography dataset (single rotation axis, parallel-beam illumination) are distributed over multiple nodes in a computing cluster. Splitting the volumes into thick “slabs,” each consisting of a stack of slices perpendicular to the rotation axis, the areas on the detector influenced by the slabs are all disjoint. This allows treating the slabs as independent volumes in the reconstruction, where each node is responsible for a specific part of the 3D volume (its slab) and a specific part of the projection data. Figure 2 illustrates the situation for a circular cone-beam acquisition scheme. Due to the divergence of the beam, each line from source to detector intersects with multiple slices perpendicular to the beam. As a result, the areas on the detector that are influenced by each slab are overlapping. To perform a *forward projection* (computing the projections of a given 3D volume) where each node is responsible for one slab, the computational results for adjacent slabs have to be merged to form the projections in these overlapping regions. This introduces the need for network communication between the nodes, which is typically much slower than memory access within the nodes. Moreover, such communication typically requires low-level network programming using the message-passing interface (MPI) or other message-passing libraries, which can turn elegant high-level implementations of reconstruction algorithms into technically complex programmes that are tied to particular computing architectures.

Our goal for the work presented here is to create a software platform that allows for easy implementation of advanced reconstruction algorithms in a non-slice-based setting, that is scalable from a single workstation to a medium-sized computing cluster. By focusing on a more generic geometry model, our approach can alleviate all of the drawbacks of a slice-based approach mentioned above: (1) it provides the ability to perform large-scale (up to a TB of data size or more) reconstructions that can be used in both parallel-beam and circular cone-beam setups; (2) it allows for the implementation of spatial priors that exploit the 3D dependencies between the information present in consecutive slices.

Our platform is an extension to the ASTRA toolbox [15], a toolbox for rapid implementation of advanced tomography algorithms that offers a high-level mathematical syntax for expressing the algorithms, while performing the basic computational operations using an optimized parallel GPU-implementation. The ASTRA toolbox offers a high degree of geometrical flexibility, making it possible to use the same algorithms for different geometrical setups [16, 17]. Without our new extension, the ASTRA toolbox is limited to the processing of 3D volumes that fit fully into the available system memory of a single workstation. Our distributed computing extension makes it possible to use the same high-level model for specifying algorithms, while the algorithm can be carried out in a distributed computing environment, with limited overhead for communication between the compute nodes.

**Fig. 3:**
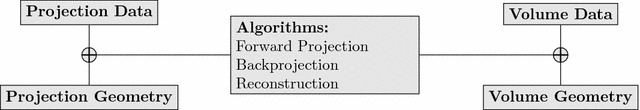
Relationships between the core ASTRA concepts

**Fig. 4:**
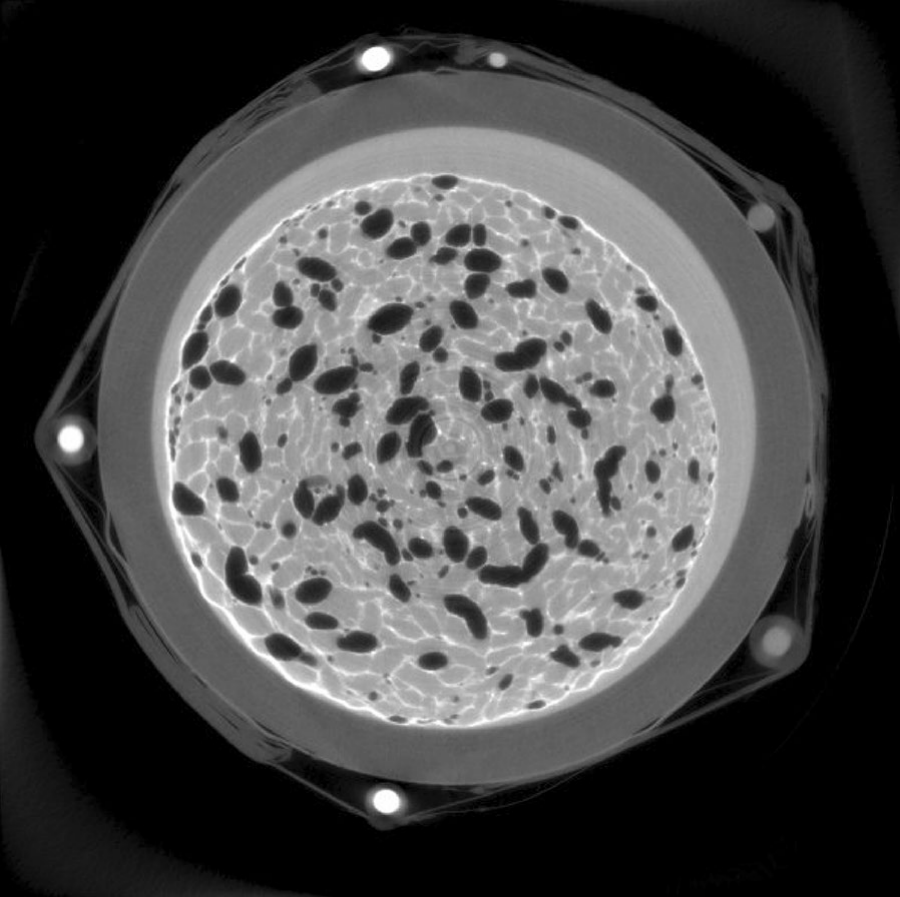
SIRT reconstruction of tissue engineering scaffold. Reconstruction with 150 iterations of SIRT on 20 GPUs of an alginate/hydroxyapatite bone tissue engineering scaffold. Courtesy of Dr. Francesco Brun and Dr. Gianluca Turco, University of Trieste, Italy

A key challenge in the design of such a distributed computing framework is hiding the details of computing and memory synchronization for the user. We use Python as the language for specifying the reconstruction algorithms. By using the capabilities of the Python language for code serialization and remote execution, the user can provide a single algorithm implementation that looks almost identical to a standard single-node algorithm. Operations performed on large volumes are carried out by the individual nodes without the need for unnecessary expensive data-communication. The forward and backprojection operations, which are usually the most time-consuming, are also carried out in a distributed way, synchronizing only the memory overlap between detector regions that reside on different nodes.

This paper is structured as follows: In “Methods” section, we describe our approach for distributing both the 3D volume data and the projection data across multiple nodes in a cluster, where each node is responsible for processing only part of the data. We then describe the various operations that are supported in our framework: Forward projection, backprojection, and voxel-based operations on the 3D volume. “Usage” section then covers the high-level usage of our platform and illustrates its use by a concrete example, where the CGLS algorithm is combined with a smoothness prior in the volume domain. In “Results” section, we present timing results that demonstrate the scalability of our approach, report on the subtle differences that can arise between the results of a distributed reconstruction as compared to a reconstruction on a single node, and show reconstructions of both simulated and experimental data, followed by “Discussion and conclusions” section.

## Methods

To facilitate re-use of code, and hide as many distributed programming details as possible, we have chosen to keep the interface similar to single-node usage of ASTRA. We have therefore made distributed ASTRA still execute a single Python script on a single master node. The ASTRA functions called by this script then internally manage the other nodes and distribute the work to these nodes. For the communication between nodes we use MPI. In this section, we describe the distributed operations in more detail.

**Fig. 5:**
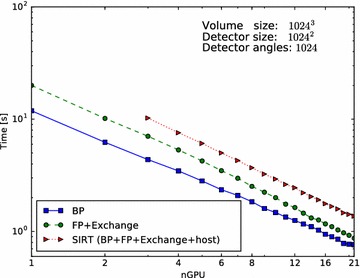
Parallel-beam performance for different GPU counts. Performance scaling of parallel-beam BP, FP and SIRT routines from 1 to 21 GPUs. Presented is the time required, in seconds, to execute a single BP (*solid line*, *square*), single FP (*dashed line*, *circle*) and single SIRT iteration (*dotted line*, *triangle*). Missing data points are due to not enough total GPU memory

**Fig. 6:**
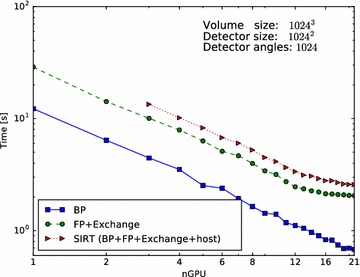
Cone-beam performance for different GPU counts. Performance scaling of cone-beam BP, FP and SIRT routines from 1 to 21 GPUs. Presented is the time required, in seconds, to execute a single BP (*solid line*, *square*), single FP (*dashed line*, *circle*) and single SIRT iteration (*dotted line*, *triangle*). Missing data points are due to not enough total GPU memory

**Fig. 7:**
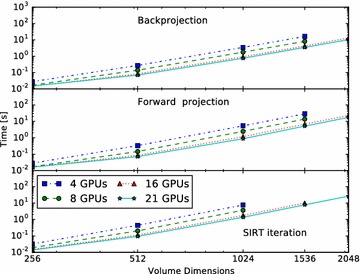
Parallel-beam performance for different volume sizes. Performance scaling of parallel-beam BP, FP and SIRT routines over a range of volume sizes. Presented is the time required, in seconds, to execute a single BP (*top panel*), single FP (*bottom panel*) and single SIRT iteration (*middle panel*). We increase the volume size from $256^3$ to $2048^3$. For a volume size of $N^3$, the detector size is $N^2$, and *N* projections are used. Missing data points are due to not enough total GPU memory

First, we summarize the use of the ASTRA toolbox from Python on a single node here. As we illustrate in Fig. 3, both input and output data are stored internally in *data objects*, as single precision floating point. These come in two types: *projection data*, and *volume data*. Associated to these objects are, respectively, a *projection geometry* and a *volume geometry*. These describe the geometry of the experimental setup, with the position and movement of the X-ray source (or the direction of the rays), the number and size of pixels in the detector, and the number and size of voxels in the reconstruction volume. On these data objects, users can call *algorithms*, such as the Forward Projection or Backprojection operators, or reconstruction algorithms including, but not limited to, filtered backprojection (FBP), Feldkamp-Davis-Kress (FDK), and the simultaneous iterative reconstruction technique (SIRT). These concepts and functions are demonstrated in the sample Python code in Table 1.

**Table 1:**
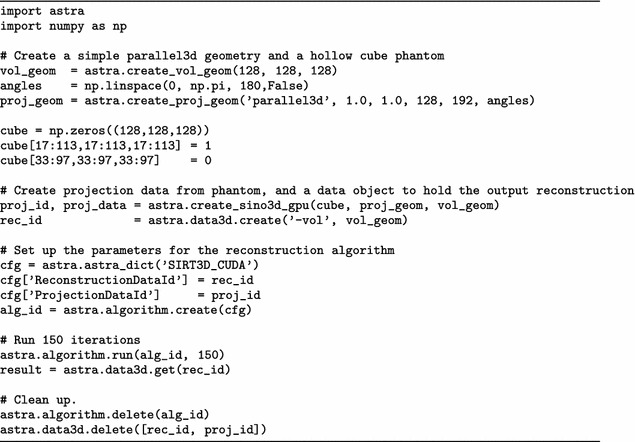
Calling single-node GPU SIRT

### Distribution of data

To go beyond the use of a single node, we have to distribute the data objects over multiple nodes. For this distribution, we make a distinction between volume data and projection data. For efficiency reasons, we assume that we have a setup that approximately rotates around the *z*-axis, with either a rotating sample or rotating source and detector.

Suppose we have *N* nodes. First of all, we split the volume into *N* independent sub-volume blocks of approximately equal size, where each node is assigned as a different set of slices orthogonal to the *z*-axis, which we call a “slab.” Next, we compute for each such volume slab the projection extent on the detector, combined for the full range of projection angles; this is the region of the detector that is affected by an FP of the slab (in any projection direction), or, equivalently, the region of the detector that affects a BP to the slab. Note that, the detector regions corresponding to different volume slabs can overlap, cf. Fig. 2.

Each node stores the data for its volume slab, and the data for the corresponding detector region. In this way, we store a limited amount of data on each node, while also only requiring a limited amount of communication between neighbouring nodes for the FP and BP operations, as we will describe in the subsection on these operations below.

### Ghost cells

The domain decomposition splits the volumes such that the amount of data in the nodes is minimized and each node therefore only holds the data that are necessary for the FP and BP operations. However, certain operations, such as computing gradients, or applying image filters, require information from (usually a small set of) neighbouring voxels. When all voxels are in the same memory buffer, this is not a problem and the required data can be read directly. However, when the neighbouring voxels are stored in the memory of another node, this would no longer be possible. To enable the execution of these operations, we have the option to make the domains, as computed by our domain decomposition, slightly larger than otherwise strictly necessary. These extra slices which overlap with neighbouring nodes, we call ghost cells. They are automatically synchronized after FP and BP operations.

The addition of ghost cells allows users to execute their multi-voxel operations as before, without having to worry about the fact that they are applied to a subset of the full dataset. The toolbox contains utility functions to automatically select the unique subset within the local volume in case the user has to perform operations on unique elements only (e.g. compute a norm or inner product).

### Forward projection and backprojection

Computing the result of an FP operation on the overlapping regions on the detector requires volume data from multiple nodes. Since FP as modelled by ASTRA is a linear operation, we can perform the FP operation for each node separately, and afterwards sum the results in the overlapping detector regions by exchanging data between nodes. This is achieved using the overlap configuration computed during the domain decomposition. These overlapping slices are exchanged with the neighbouring processes, and the overlapping detector regions are combined. By exactly computing the domain extents, we minimize the amount of data that have to be exchanged, while ensuring that afterwards, each node has a consistent and correct copy of its detector region.

**Fig. 8:**
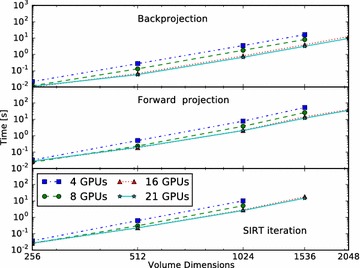
Cone-beam performance for different volume sizes. Performance scaling of cone-beam BP, FP and SIRT routines over a range of volume sizes. Presented is the time required, in seconds, to execute a single BP (*top panel*), single FP (*bottom panel*) and single SIRT iteration (*middle panel*). We increase the volume size from $256^3$ to $2048^3$. For a volume size of $N^3$, the detector size is $N^2$, and *N* projections are used. Missing data points are due to not enough total GPU memory

**Fig. 9:**
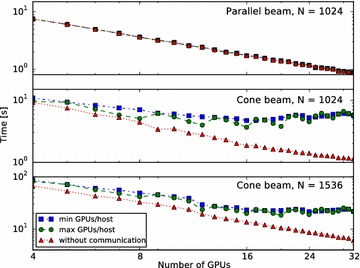
Effects of distribution of GPUs over nodes. Performance scaling of SIRT routines over a range of GPU counts on a cluster of 8 hosts with 4 GPUs each, with two different ways of distributing the GPUs over hosts: with “max GPUs/host” we cluster the GPUs as much as possible on hosts, while with “min GPUs/host” we use as few GPUs per host as possible. The time “without communication” is the time spent on actual computation, with communication between nodes disabled. The volume size is $N^3$, the detector size is $N^2$, and *N* projections are used. The time shown is the time for a single SIRT iteration

**Fig. 10:**
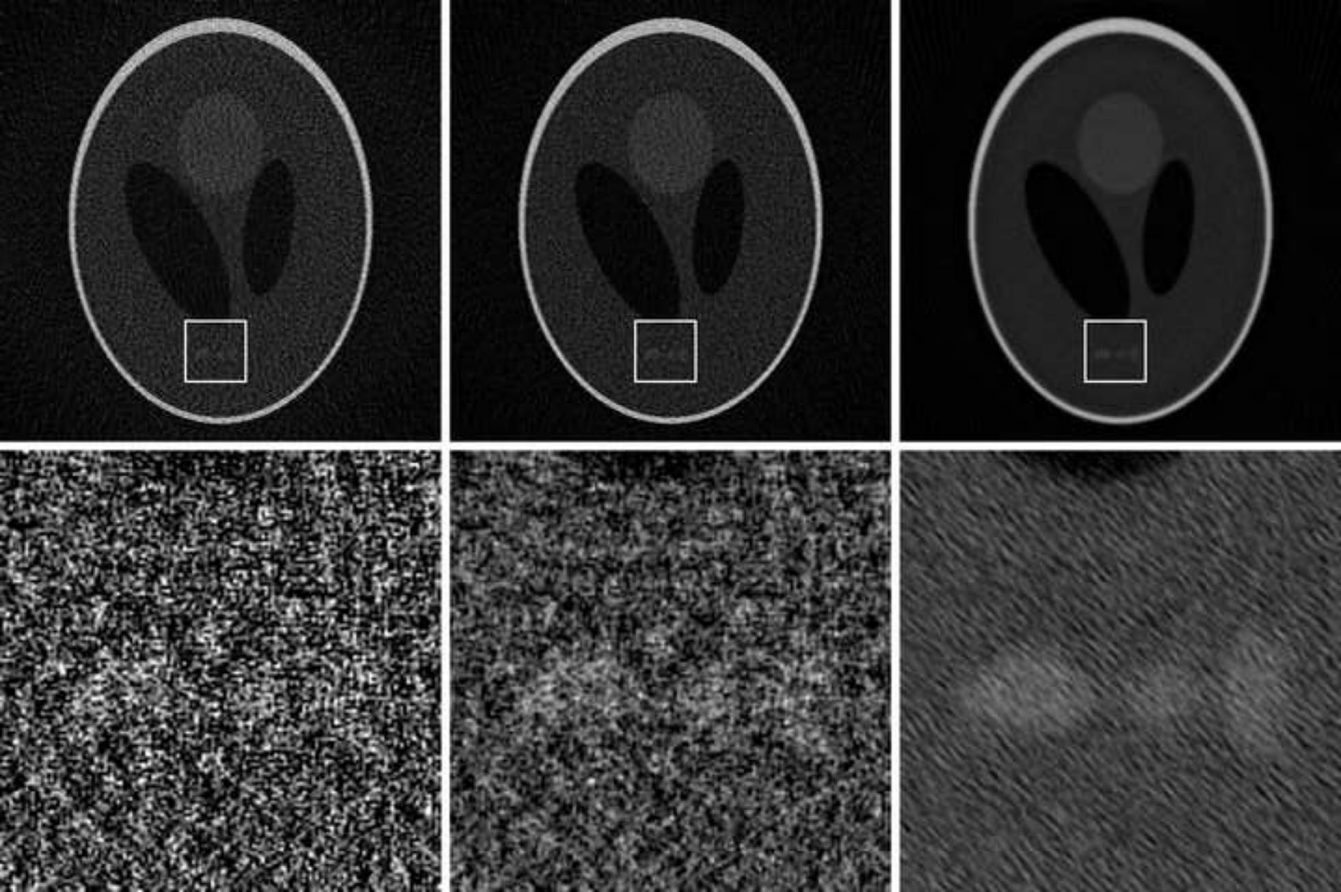
CGLS + Sobolev reconstructions. Slices of reconstructions using the described CGLS + Sobolev algorithm, with Sobolev weights set to 0, 10, 100, from left to right. Below are magnified versions of a small region of the slices to more clearly see the effect on noise and features. The projection data consisted of 180 simulated noisy cone-beam projections of $1024^2$ pixels, with a reconstruction volume of $1024^3$ voxels

**Fig. 11:**
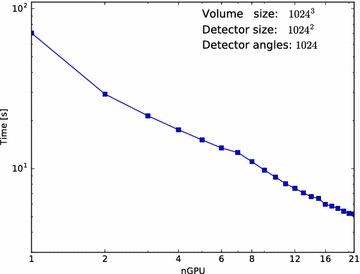
CGLS + Sobolev multi-node performance. Average execution time per iteration of the described CGLS+Sobolev algorithm, with as input 1024 projections of size $1024^2$ and an output volume of $1024^3$

For the backprojection operation, each node locally stores the part of the detector data needed to perform a BP operation, so this can be performed locally and independently on each node.

### Other operations

All iterative reconstruction algorithms need intermediate operations apart from the FP and BP steps. These include (but are not limited to) basic arithmetic on the data volumes, image filtering steps such as blurring or computing gradients, reduction operations such as norms or inner products, and reading and writing data to disc.

Some of these operations are available directly using utility functions provided by the ASTRA toolbox, a number of which are shown below in “Usage” section. Others can be implemented using a provided general method to execute a custom written Python function across all nodes. With this functionality, the user can perform custom operations on the distributed dataset, thereby taking full advantage of the extra available computation power when using multiple nodes.

Using Python’s functionality to serialize code, the user-supplied custom function is sent from the master script to all nodes, and executed on each node. There, the function can access the local data on each node, and perform the required functions on that data.

The user-supplied function can choose to either process all local data on a node, or only to process data for which the current node has the authoritative copy. This last functionality can for example be used to compute dot products, where it is important not to perform computations twice on overlapping regions. After any such operations, the ASTRA toolbox can synchronize all data on the nodes again, to propagate any changes to the overlapping regions.

In “Usage” section, we show two basic functions that process distributed volumes in this way.

### SIRT

We have extended the GPU implementation of the Simultaneous Iterative Reconstruction Technique (SIRT) [18] in ASTRA to this MPI framework, using the distributed FP and BP operations described above, and also performing all intermediate arithmetic directly on the GPUs.

During an iteration of SIRT, the only communication between nodes takes place at the end of the FP operation as described above. The BP operation requires no additional communication, and neither do all other arithmetic operations, which are performed locally on each node.

## Usage

### Launching code

The distributed code is integrated in the Python bindings of the ASTRA toolbox, which allows near-transparent use for the user of the distributed toolbox functions. All the functions that handle data and execute functions have been made MPI aware and will handle the distribution and gathering of data. This functionality is enabled by a special launcher. This launcher programme will start clients on all nodes, and then executes the user’s script on the master node. A launch could look as follows:
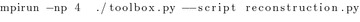


This will use four nodes to run a script called reconstruction.py written by the user. As mentioned before, the user script itself is executed only on a single master node, but supported ASTRA calls will use all four nodes.

A distributed version of the non-distributed script given before in Table 1 is presented in Table 2. It differs from the single-node script only in the single line calling mpi.create that enables the distributed functionality when combined with mpirun and toolbox.py.

**Table 2:**
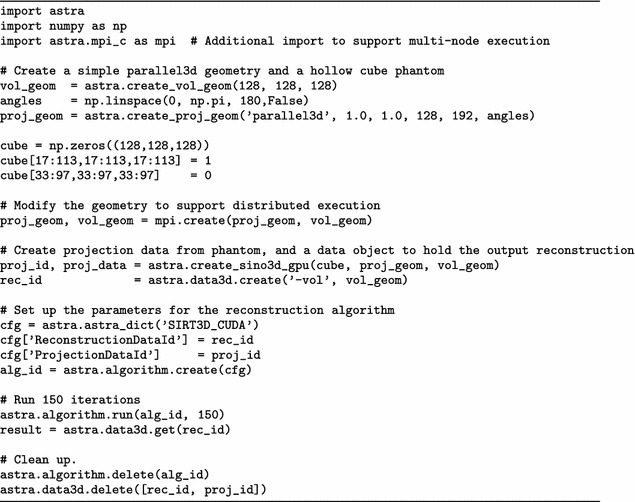
Calling distributed SIRT

### User-supplied functions

We present two examples, Tables 3 and 4, to illustrate the functionality to run user-supplied functions on distributed data volumes.

**Table 3:**
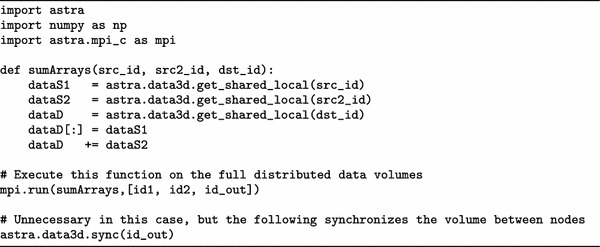
Running a custom function on distributed data objects

**Table 4:**
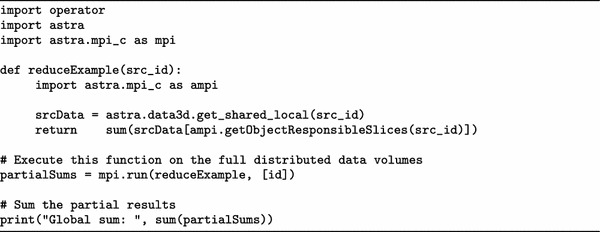
Running a custom reduction function on distributed data objects

The first example, Table 3, adds two data volumes (pointwise) and stores the sum in a third data volume. This is implemented by a function called sumArrays which performs the actual addition using NumPy arrays. It takes ASTRA object IDs as input, and accesses the data contained in the objects using the get_shared_local function.

To execute this function on all the available processes, the function mpi.run is called, with as arguments the function to be distributed, and a list of parameters to be passed to the function.

Finally, the last line could be used to synchronize any overlapping regions. However, since the function sumArrays keeps all data consistent, there is no need to call that in this specific example.

Certain operations should solely be executed on unique data. For example, when computing the inner product of a volume the overlapping regions of the volumes should not be included. The content of these regions is available on multiple processes and would therefore be added multiple times. To exclude this overlap in the computations there is a function that selects the data for which the current node has the authoritative copy, which we refer to as the slices that the current node is responsible for. The usage of this function is presented in Table 4 using a simple sum example. The function reduceExample calls getObjectResponsibleSlices to obtain the necessary subset of the data, sums this, and returns the partial local sum. The function mpi.run returns a list containing these partial local sums from all nodes, and we sum these values to obtain the full sum of the data volume.

### A sample reconstruction algorithm

Finally, we show an implementation of a full iterative reconstruction algorithm using the distributed functionality of ASTRA presented in this paper.

We make no claims here on the suitability of this algorithm for reconstruction of specific projection datasets, but use it to illustrate a set of operations used in many algorithms.

Writing *x* for an (unknown) volume (in vector form), *p* for the measured projection data (also in vector form), and *W* for the tomographic system matrix, a basic algebraic formulation for the tomography problem is given by

$$\begin{aligned} \min _x ||Wx - p ||_2^2. \end{aligned}$$

To this, we add a regularization term with the $\ell _2$-norm of the discrete gradient (Sobolev prior) of the image, denoted by $||\nabla x ||_2$, and with $\lambda ^2$ as the weight of this term:

$$\begin{aligned} \min _x ||Wx - p ||_2^2 + \lambda ^2||\nabla x ||_2^2. \end{aligned}$$

Since both *W* and $\nabla$ are linear operations, we can stack these operators into a single operator to obtain

$$\begin{aligned} ||Wx - p ||_2^2 + \lambda ^2 ||\nabla x ||_2^2 = \left|\left|\left( {\begin{array}{c}W\\ \lambda \nabla \end{array}}\right) x - \left( {\begin{array}{c}p\\ 0\end{array}}\right) \right|\right|_2^2. \end{aligned}$$

Our sample script in Table 5 implements the conjugate gradients least-squares (CGLS) algorithm [19] for this stacked operator, which we denote by $$A = \left( {\begin{array}{c}W\\ \lambda \nabla \end{array}}\right)$$.

**Table 5:**
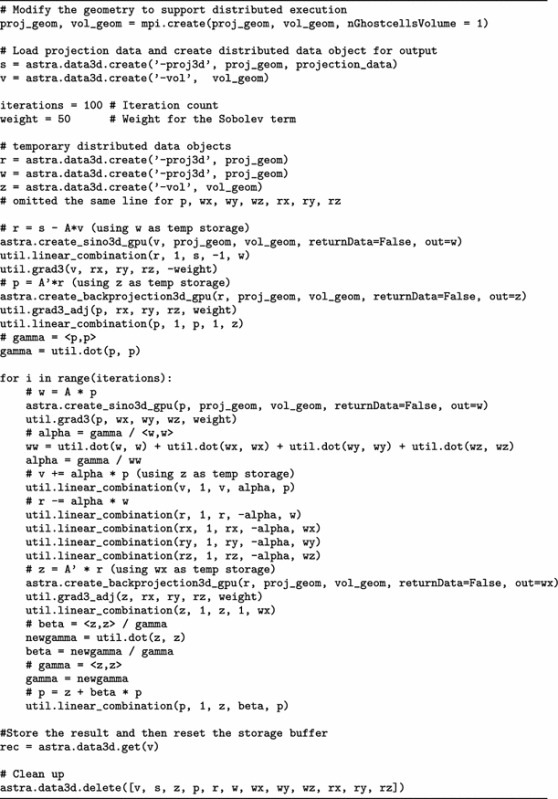
ASTRA/MPI implementation of CGLS with Sobolev regularization

It calls the FP and BP operators (corresponding to multiplication with *W* and $W^T$, respectively) using the ASTRA functions create_sino3d_gpu and create_backprojection3d_gpu. It also calls utility functions grad3 and grad3_adj to perform the $\nabla$ and $\nabla ^T$ operations, respectively.

The function dot is used to compute inner products, and finally, the script uses the linear_combination utility function to compute various linear combinations of pairs of vectors, as described in the comments in the script.

In “Results” section below, we show sample reconstructions and timings for this implementation.

## Results

To demonstrate the MPI implementation of ASTRA described in this paper is able to produce proper reconstructions of real-world tomographic data, we have run 150 iterations of the SIRT algorithm on projection data of an alginate/hydroxyapatite bone tissue engineering scaffold [20]. The data consist of 1800 projections of $2005\times 1335$ with a cone angle of approximately $15.8^{\circ}$, and the reconstruction volume is $1984\times 1984\times 1332$. We used 20 GPUs for this reconstruction. A representative slice of the reconstructed volume is shown in Fig. 4.

To further validate the MPI implementation, we have compared the results of multi-node runs of the FP, BP and SIRT (50 iterations) functions against the existing single-node implementations in ASTRA. We have used a $512^3$ volume with 180 projections of $512^2$ for this, and 2, 3, 4, and 16 nodes.

The results are summarized in Table 6. As expected, the parallel-beam results are identical (to full machine precision), as there is no need for communication between nodes. For cone beam, there are small differences. These are caused by small numerical inaccuracies during the tracing of rays, which differ between tracing through the subvolume on each individual node compared to tracing through the full volume. However, the differences are isolated, and the average error remains very small.

**Table 6:** Comparison of multi-node with single-node results

	2 Nodes	3 Nodes	4 Nodes	16 Nodes
Parallel FP	0	0	0	0
Parallel BP	0	0	0	0
Parallel SIRT	0	0	0	0
Cone FP	5.7e−6	5.5e−6	5.6e−6	5.8e−6
Cone BP	3.7e−7	3.6e−7	3.8e−7	3.8e−7
Cone SIRT	2.3e−6	2.4e−6	2.4e−6	2.4e−6

Reported is the normalized root mean squared error (NRMSE), the square root of the mean squared error divided by the maximum value of the reference output

In this section, we also show how the performance of three different methods scales with volume size and number of used nodes: a single FP (including the required communication), a single BP and the SIRT reconstruction algorithm. Each SIRT iteration consists of an FP (including communication), a BP, and auxiliary functions required for the reconstruction algorithm. For SIRT, we present the average time of a single iteration. With these methods, we have performed three different experiments. In the first experiment, we tested the multi-node scaling on a fixed sized volume using 1 to 21 GPUs. In the second experiment, we scale the volume size from 256 to 2048 and measured the time that each method takes using 4, 8, 16, and 21 GPUs. In the third experiment, we use a different cluster (with more nodes) to determine how the distribution of GPUs over nodes affects performance.

For all computational experiments, we used a cubic reconstruction volume of size $N^3$, with *N* projections with a square detector of size $N^2$. For the cone-beam experiments, we have used a cone angle of approximately $7.8^{\circ}$.

The cluster we used for the first two experiments consists of three servers, connected using 100 Gbit EDR Infiniband cards and an EDR Infiniband router. Each server has an Intel Xeon E5-2698 CPU, 128 GB of RAM and contains 7 Titan X (Maxwell) GPUs from NVIDIA with 12 GB of RAM each. For these tests, boost was disabled and the GPUs were manually set to their maximum supported clock speed. The CPU has two 16 lane PCIe slots available. Since this is not enough for the available devices, there are PCIe switches in between the PCIe devices and the CPU. Each switch has 16 PCIe lanes to the CPU and 64 lanes for the connected devices. The first switch holds 4 GPUs, so if all these GPUs communicate with the CPU at the same time, then this results in a 4:1 bottleneck. The second switch holds 3 GPUs and the Infiniband card. The servers are running Ubuntu Linux 16.04, with CUDA 7.5, and gcc 4.8.4.

This cluster allows us to scale from 1 to 21 GPUs. We always fill a single node before we add a second node. For example, with 7 GPUs a single machine is used, and with 8 GPUs two machines are used with 7 processes on the first and 1 process on the second node.

The results of the first experiment are presented in Fig. 5 (parallel beam) and Fig. 6 (cone beam), for the case $N=1024$. On the horizontal axis, we indicate the number of GPUs and on the vertical axis the time it takes to complete one BP (solid line), FP (dashed line) or one SIRT (dotted line) iteration.

For parallel beam, everything scales linearly as there is nearly no communication overhead.

For cone beam, the BP scales nearly linearly from 1 to 21 GPUs as there is no communication required and the sub-volumes are large enough to saturate the GPU. For the FP the scaling is affected by network communication. We can see that the scaling is less ideal than that of the BP. But although the network communication negatively impacts the scaling, the execution time keeps decreasing when more GPUs are added. The SIRT iteration, which consists of both an FP, BP, network communication and host operations, also benefits from using more GPUs and continues to scale. As with the FP operation, we see the influence of network communication, but here the effect of adding GPUs becomes negligible when using 16 GPUs. With 17 or more GPUs, we hardly see any improvement in the execution time as it is dominated by the communication time. The more GPUs are used, the smaller the blocks per GPU and the lower the computation time, but the number of slices that overlap will form a larger fraction of the total block size on a GPU. So with more GPUs, we have to exchange relatively more data with more neighbours while the GPU has less data to process. If we were to increase the number of GPUs further beyond 21, we expect the total runtime will start to increase for this volume size.

In Figs. 7 (parallel beam) and 8 (cone beam), we present the results of the second experiment. Each of the three panels shows a different operation; BP in the top, FP in the middle and SIRT in the bottom panel. For each, we present the execution time for $N=256$ up to $N=2048$ using the four different GPU configurations. Ignoring communication, it is expected that doubling *N* results roughly in a 16$\times$ increase in execution time. The lines for the BP match this approximately, since there is no need for communication there. For FP and SIRT, communication time becomes a smaller fraction of total execution time when the volume size increases.

For the third experiment, we have used a cluster of eight machines, each with two Intel Xeon E5-2630 CPUs, 128 GB of memory (except for the master node, which has 256 GB), and four Titan X (Maxwell) GPUs from NVIDIA with 12 GB of RAM each. These machines are connected with a Gbit ethernet network (i.e. no Infiniband). They are running Fedora Linux 24, with CUDA 8.0, and gcc 5.4.0.

In Fig. 9, we present the results. They are divided into three configurations. All of these use a volume of $N^3$ and *N* projections of size $N^2$. The configurations are parallel beam with $N=1024$, cone beam with $N=1024$, and cone beam with $N=1536$. As before, the cone angle is approximately $7.8^{\circ}$. For each configuration, we have varied the number of GPUs, and distributed these GPUs over the hosts in two different ways: either filling up a host completely before moving to the next one as in the first two experiments (labelled “max GPUs/host” in the figure), or using as few GPUs per host as possible (labelled “min GPUs/host”). Additionally, the points labelled “without communication” show the time spent on computation without communication between GPUs, which we have determined by disabling the exchange of overlapping regions in the MPI SIRT implementation described. The jump observed between 11 and 12 GPUs with the $1536^3$ cone-beam configuration is due to the fact that with 12 GPUs, all temporary volumes used by SIRT fit entirely in the available GPU memory. With fewer GPUs, temporary volumes are stored in host memory, and computations other than FP and BP are performed by the CPU. (This is not an issue for the $1024^3$ volumes.) Clustering as many GPUs together as possible leads to higher performance on this cluster since fewer communication channels traverse the network. When compared to the first experiment, the effect of the slower network of this cluster can be seen.

Finally, in Fig. 10, we show slices from three reconstructions using the Sobolev–regularized CGLS algorithm implemented in Table 6. We have simulated projection data consisting of 180 projection of $1024^2$ pixels of a 3D variant of the Shepp-Logan phantom, with a fairly high level of Poisson noise. We have run 100 iterations, using a reconstruction volume of $1024^3$ voxels, with the Sobolev term weighted with three different weights: $\lambda = 0$, 10 and 100. The figure shows the central slices of these three reconstructions. The effect of a stronger weight on the Sobolev term is clearly visible.

To show the scaling of performance, we have run this algorithm on a larger dataset of 1024 projections of $1024^2$ on 1–21 GPUs. The average time per iteration is shown in Fig. 11.

## Discussion and conclusions

In this paper, we have presented the Distributed ASTRA toolbox, which offers computational building blocks for implementing tomography algorithms that are scalable from a single GPU-equipped workstation to a moderately sized cluster of GPU-equipped nodes. Our work extends the functionality of the existing ASTRA Tomography toolbox by allowing efficient reconstructions of volumes that do not fit in the memory of a single GPU, on either a single node or using multiple nodes of a GPU cluster. We have shown that the method scales to at least a volume size of $N=2048$ using 21 GPUs. Similar to the current operations implemented in the ASTRA toolbox, our work will enable the rapid design and implementation of distributed advanced reconstruction algorithms, using the distributed FP, BP and SIRT implementations as building blocks.

Through its design, the distributed ASTRA toolbox allows implementing algorithms in Python using a high-level syntax that is close to the formal mathematical algorithm description, while performing the distribution of data and computation in a way that is almost hidden from the user. By keeping the data on the individual nodes as much as possible and only exchanging the parts of 3D volume and projection data at the boundaries between the 3D slabs, communication between the nodes is minimized.

The experiments from “Results” section indicate that the implemented parallel distribution method scales well for practical volume sizes and GPU counts. The larger the volume, the more GPUs can be used before communication overhead prevents a speedup from adding additional GPUs. Yet there is still room for improvement. In particular, better scaling might be achieved when performing the exchange of the overlap regions in parallel with computation, rather than sequentially.

At present, our implementation is limited to single-axis tomography acquisition schemes that are close to the parallel-beam or circular cone-beam geometry, and assumes a homogeneous cluster with similar GPUs and nodes. For these configurations, a uniform slab-based distribution of the data is highly appropriate. For more general acquisition schemes however, such as helical cone-beam acquisition and laminography, or for heterogeneous clusters, the way the data are distributed across the nodes will have to be adapted to achieve reasonable computational performance. Our current research focuses on the development of more automatic ways of performing the data distribution that can deal with more general acquisition geometries.
